# Pneumococcal Sepsis Revealing Pediatric Systemic Lupus Erythematosus with Sjögren’s Syndrome Overlap: A Case Report

**DOI:** 10.3390/pediatric18020051

**Published:** 2026-04-02

**Authors:** Francesco Accomando, Vittorio Albertazzi, Francesco Girelli, Michela Biscarini, Melodie O. Aricò, Enrico Valletta

**Affiliations:** 1 Department of Pediatrics, G.B. Morgagni–L. Pierantoni Hospital, Azienda Unità Sanitaria Locale Romagna, 47121 Forlì, Italyenrico.valletta@auslromagna.it (E.V.); 2 Department of Nephrology and Dialysis, G.B. Morgagni–L. Pierantoni Hospital, Azienda Unità Sanitarlia Locale Romagna, 47121 Forlì, Italy; 3 Rheumatology Unit, Department of Medicine, G.B. Morgagni–L. Pierantoni Hospital, Azienda Unità Sanitaria Locale Romagna, 47121 Forlì, Italy; 4 Department of Radiology, G.B. Morgagni–L. Pierantoni Hospital, Azienda Unità Sanitaria Locale Romagna, 47121 Forlì, Italy

**Keywords:** children, systemic lupus erythematosus, Sjögren’s syndrome, pneumococcal sepsis

## Abstract

Background: Systemic lupus erythematosus (SLE) may present with heterogeneous clinical manifestations in pediatric patients. Although infections are a major cause of morbidity and mortality in SLE, severe bacterial infections rarely represent the presenting clinical event leading to diagnosis. Case description: We report the case of a 13-year-old boy diagnosed with SLE with Sjögren’s syndrome overlap who presented with pneumococcal sepsis. The patient was admitted with high-grade fever and facial swelling, and blood cultures grew *Streptococcus pneumoniae*. Although an initial clinical response to antibiotic therapy was observed, fever subsequently recurred, accompanied by persistent systemic symptoms and progressive laboratory abnormalities. Further investigations revealed hematologic abnormalities, serosal involvement, renal disease, and a characteristic autoantibody profile. The patient fulfilled the 2019 ACR/EULAR classification criteria for SLE after comprehensive autoimmune evaluation. The overlap with Sjögren’s syndrome was supported by the autoantibody profile and imaging findings involving the parotid glands. Following treatment with intravenous methylprednisolone pulses, oral prednisone, hydroxychloroquine, and mycophenolate mofetil, the patient showed rapid clinical improvement and sustained remission. Conclusions: This case highlights that severe invasive bacterial infection may occasionally be the clinical circumstance that leads to the diagnosis of pediatric systemic lupus erythematosus. Persistent systemic inflammation or evolving multisystem involvement despite appropriate antimicrobial therapy should prompt consideration of an underlying autoimmune disease, even in patients without a prior history of immune dysfunction.

## 1. Introduction

Pediatric-onset systemic lupus erythematosus (SLE) accounts for approximately 20% of all cases and is often associated with a more severe disease course than adult-onset SLE, with higher rates of renal, hematologic, and neuropsychiatric involvement [1]. Overlap syndromes represent a diagnostic and therapeutic challenge in pediatric rheumatology, and the coexistence of SLE and Sjögren’s syndrome (SS) has rarely been reported in children [2]. Infections are among the leading causes of morbidity and mortality in patients with SLE [3]. Although infections frequently complicate the disease course, severe bacterial infections as the presenting clinical event leading to the diagnosis of SLE are exceedingly rare and pose significant diagnostic challenges [4,5]. Here, we describe a pediatric case in which invasive pneumococcal infection was the presenting clinical event that led to the diagnosis of SLE with Sjögren’s syndrome overlap.

## 2. Case Report

A 13-year-old boy of Nigerian origin was admitted with a 2-day history of high-grade fever (maximum temperature, 40 °C) associated with right facial and lip swelling. Initial laboratory investigations revealed anemia, thrombocytopenia, and lymphopenia (white blood cells 8670/mm^3^; neutrophils 7250/mm^3^; lymphocytes 900/mm^3^; hemoglobin 10.7 g/dL; platelets 80,000/mm^3^), together with markedly elevated inflammatory markers (C-reactive protein 135.9 mg/L). Empiric intravenous ceftriaxone was initiated, resulting in defervescence within 24 h.

The patient had no prior history of chronic illness and had not received immunosuppressive therapy. There was no documented history of recurrent parotid swelling, sicca symptoms, or other features suggestive of sialadenitis before this acute presentation. Family history was negative for autoimmune diseases or primary immunodeficiency disorders, and the parents were non-consanguineous. Vaccinations had been administered according to the national childhood immunization schedule; pneumococcal vaccination had not been performed. HIV serology was negative. Serum immunoglobulin levels, including IgG subclasses, and peripheral lymphocyte subset analysis were within normal limits. Given the patient’s previously unremarkable clinical history, with no recurrent or severe infections and no features suggestive of an underlying immune disorder, an extended immunological workup was not performed during the acute phase.

**Figure 1:**
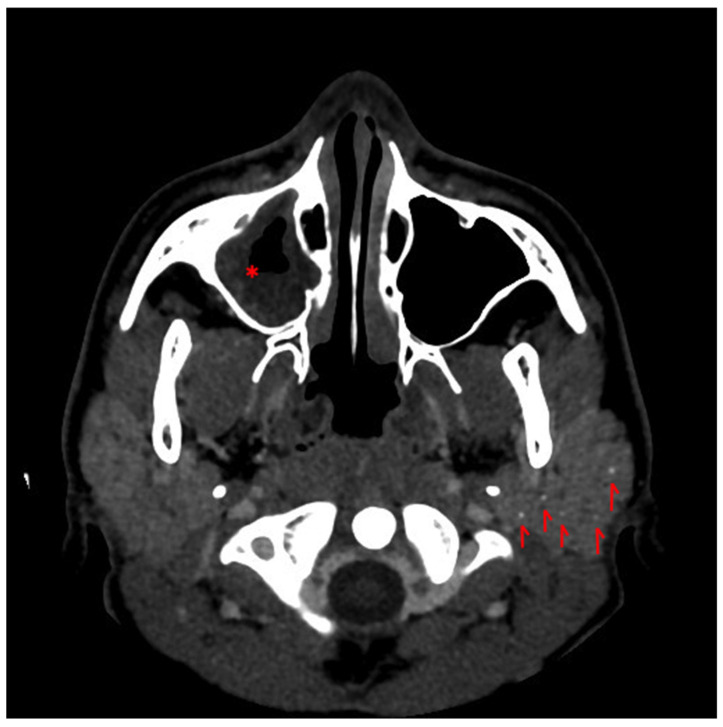
Contrast-enhanced CT of the facial soft tissues demonstrating bilateral enlargement of the parotid glands with heterogeneous hypodense parenchyma and intraparenchymal microcalcifications (arrows), findings compatible with inflammatory glandular involvement. Circumferential soft-tissue density occupying the right maxillary sinus (*), consistent with inflammatory sinus disease.

Chest radiography was negative for pulmonary infiltrates or pleural effusion. Initial echocardiography was unremarkable, with no evidence of endocardial involvement or pericardial effusion. Abdominal ultrasonography was negative for focal infectious findings; the spleen was normal in size (bipolar diameter 11.8 cm) and showed homogeneous echotexture. Blood cultures obtained prior to antibiotic administration grew *Streptococcus pneumoniae* (serotype 13 V). Computed tomography (CT) of the facial soft tissues and brain, performed because of persistent facial edema, demonstrated mild bilateral enlargement of the parotid glands with heterogeneous hypodense parenchyma and multiple small intraparenchymal calcifications. Bilateral cervical and submental lymphadenopathy was also present. In addition, mucosal thickening of the frontal sinuses, predominantly on the right side, and material within the right maxillary sinus were noted (Figure 1).

Four days after the initiation of antibiotic therapy, C-reactive protein levels markedly decreased (29 mg/L), whereas erythrocyte sedimentation rate remained persistently elevated (up to 89 mm/h). One week after admission, the patient experienced a recurrence of high-grade fever (up to 40.6 °C). Repeat blood cultures, laboratory tests, respiratory viral screening, Mantoux testing, galactomannan assay, and malaria testing were all negative. After three days of persistent fever, broad-spectrum antibiotic therapy with piperacillin–tazobactam and clindamycin was initiated, and antifungal coverage with fluconazole was added.

Given concerns for refractory sinusitis, a repeat CT scan of the facial skeleton was performed and showed no significant improvement. The patient subsequently underwent functional endoscopic sinus surgery (FESS); intraoperative findings revealed no purulent material, and microbiological cultures were negative. Overall, the episode was consistent with pneumococcal bloodstream infection without a clearly documented focal source. During the second febrile episode, peripheral blood smear examination revealed hyposegmented neutrophils with a Pelger–Huët-like appearance, a finding not previously observed.

A repeat echocardiogram excluded infective endocarditis but demonstrated a mild, hemodynamically insignificant pericardial effusion. In the following days, minimal bilateral pleural and abdominal effusions were detected. Bone marrow aspiration was performed to exclude an underlying hematologic malignancy and revealed a hypocellular marrow. At that stage, the cytopenias were initially interpreted in the differential diagnosis as possibly related to severe infection-associated inflammation and/or transient bone marrow suppression; however, their persistence and subsequent association with Coombs-positive anemia, serosal involvement, proteinuria, and a characteristic autoantibody profile progressively raised suspicion of an underlying systemic autoimmune disease. Urinalysis showed mild proteinuria, confirmed by a 24 h urine collection (0.68 g/24 h). Serum creatinine and estimated glomerular filtration rate were within the normal range, and blood pressure was normal for age. During hospitalization, hemoglobin progressively declined to a nadir of 8.5 g/dL. A positive direct antiglobulin (Coombs) test, mildly increased LDH levels (peak 463 U/L), normal bilirubin, and low-normal haptoglobin (0.35 g/L; lower limit of normal, 0.30 g/L) suggested possible autoimmune hemolysis. Complement levels showed a transient reduction at admission (C3 0.64 g/L, C4 0.08 g/L), with subsequent normalization. Shortly thereafter, during hospitalization, the patient developed an erythematous malar rash with a butterfly distribution, further raising suspicion of an underlying systemic autoimmune disease. No history or clinical evidence of Raynaud phenomenon was observed.

With the evolution of the clinical picture, including persistent systemic inflammation and emerging features of systemic autoimmunity, an autoimmune workup was performed. Antiphospholipid antibody testing revealed lupus anticoagulant positivity, with negative anti-β2-glycoprotein I and anticardiolipin antibodies. Antinuclear antibodies were positive at a titer of 1:640 with a speckled pattern; anti-double-stranded DNA antibodies were negative. Extractable nuclear antigen testing was positive for anti-Ro52, anti-Ro60, anti-SmD, and anti-U1-RNP antibodies (Table 1). Serial hematologic, inflammatory, complement, and renal parameters during hospitalization are summarized in Table 2, whereas the autoantibody profile at disease onset is detailed in Table 1. Based on the 2019 ACR/EULAR classification criteria, the diagnosis of SLE was established [6]. Concomitant Sjögren’s syndrome overlap was considered on the basis of parotid gland involvement and a characteristic anti-SSA/Ro and anti-SSB autoantibody profile.

The patient was treated with three pulses of intravenous methylprednisolone, followed by oral prednisone at a dose of 1 mg/kg/day, with rapid clinical improvement. Given the presence of proteinuria, renal biopsy was performed and confirmed class II lupus nephritis, based on the identification of mesangial immune deposits by immunofluorescence and electron microscopy [7]. After biopsy confirmation of lupus nephritis, hydroxychloroquine and mycophenolate mofetil were introduced as maintenance therapy.

At one-year follow-up, the patient was in clinical remission, with complete resolution of systemic symptoms and sustained normalization of inflammatory and hematologic parameters. Corticosteroid therapy was progressively tapered over the first 3–4 months to a maintenance dose of 5 mg/day, while treatment with hydroxychloroquine and mycophenolate mofetil was continued. No disease flares or infectious complications were observed during follow-up.

## 3. Discussion

In our patient, invasive pneumococcal infection was the presenting clinical event that prompted further evaluation and ultimately led to the diagnosis of pediatric-onset SLE. Although increased susceptibility to infections in SLE is well recognized, including impaired host defense against encapsulated bacteria such as *Streptococcus pneumoniae*, severe invasive infections rarely precede the diagnosis of SLE and have only occasionally been reported in the literature [4,5,8]. In this setting, acute infection may induce systemic inflammation and laboratory abnormalities that obscure or mimic autoimmune disease, thereby delaying diagnosis and appropriate treatment.

The occurrence of invasive pneumococcal sepsis raised the possibility of impaired host defense mechanisms associated with SLE, including functional hyposplenism. Functional asplenia has been described in a subset of patients with SLE [9] and may predispose to severe infections caused by encapsulated bacteria. Although splenic function was not formally assessed in our patient, this mechanism may partly explain the severity of the infection observed prior to immunosuppressive therapy. Transient hypocomplementemia was also documented at presentation and may theoretically have contributed to impaired defense against encapsulated bacteria; however, it cannot be determined whether this reflected complement consumption related to active disease or an underlying complement defect. In a previously healthy child presenting with invasive pneumococcal bloodstream infection, underlying susceptibility to encapsulated bacteria should also be considered. Relevant differential diagnoses include inherited complement deficiencies, particularly involving the classical or terminal pathway, and antibody deficiencies. In our case, an underlying complement defect or genetically determined immune dysregulation could not be formally ruled out.

Severe infections may unmask previously unrecognized immune dysregulation and thereby facilitate recognition of an underlying autoimmune disease. In this case, the inflammatory burden associated with invasive pneumococcal infection may have contributed to the clinical emergence of SLE, although a direct causal relationship cannot be established.

These observations highlight the importance of preventive strategies, including vaccination and antimicrobial prophylaxis, in patients with SLE and increased susceptibility to severe infection. In this context, follow-up should also include reassessment of immunization status and consideration of vaccination against pneumococcus, Haemophilus influenzae type b, meningococcus, and seasonal influenza, in accordance with current recommendations and the patient’s immunosuppressive treatment status [10].

An additional distinctive feature of this case is the overlap between SLE and SS, which has rarely been reported in pediatric patients. SS most often occurs as a secondary condition associated with SLE and may present with subtle or absent sicca symptoms, whereas parotid gland enlargement, a characteristic autoantibody profile, and hematologic abnormalities may be more frequent and clinically relevant manifestations in pediatric patients [2].

In our patient, although Schirmer testing and minor salivary gland biopsy were not performed, Sjögren’s syndrome overlap was considered on the basis of the characteristic autoantibody profile and imaging evidence of parotid gland involvement. Multidisciplinary evaluation of computed tomography findings revealed heterogeneous hypodense parenchyma with intraparenchymal microcalcifications, features previously described in association with Sjögren’s syndrome [11]. The combination of parotid enlargement with intraparenchymal calcifications and a characteristic anti-SSA/Ro and anti-SSB autoantibody profile also suggests that autoimmune glandular involvement may have preceded the septic episode. Therefore, pneumococcal sepsis should be regarded as the first overt clinical presentation leading to diagnosis, rather than the onset of the underlying autoimmune disease itself. Taken together, these findings are highly suggestive of SLE–SS overlap in the pediatric setting, where currently available classification criteria are largely derived from adult populations and may fail to capture early or predominantly systemic disease manifestations.

This case has some limitations. The evaluation for susceptibility to invasive pneumococcal disease was not exhaustive; in particular, inherited complement deficiency could not be formally excluded. In addition, genetic testing had not been performed at the time of writing, although further genetic evaluation has been planned. Therefore, monogenic lupus or a genetically determined immune dysregulation syndrome cannot be completely excluded.

This case highlights that, in children presenting with severe invasive bacterial infection, persistent systemic inflammation or evolving multisystem involvement despite appropriate antimicrobial therapy should prompt consideration of an underlying autoimmune disorder.

## Figures and Tables

**Table 1:** Autoantibody profile at disease onset.

Autoantibody	Result	Reference Range/Cut-Off
Antinuclear antibodies (ANA, IFA)	Positive, 1:640 (speckled)	Negative; significant ≥ 1:160
Anti–double-stranded DNA (EIA)	15.0 kUI/L	Negative < 10; doubtful 10–15; positive > 15
Anti–ds DNA (*Crithidia luciliae* IFA)	Negative	Negative
Anti-Ro52 (SSA)	>240 kUI/L	Negative < 7; positive > 10
Anti-Ro60 (SSA)	>240 kUI/L	Negative < 7; positive > 10
Anti-La (SSB)	20.0 kUI/L	Negative < 7; positive > 10
Anti-SmD	330.0 kUI/L	Negative < 7; positive > 10
Anti-U1-RNP	166.0 kUI/L	Negative < 5; positive > 10
Anti-Scl-70	0.6 kUI/L	Negative < 7
Anti-Jo-1	0.2 kUI/L	Negative < 7
Anti-cardiolipin IgG	2.7 GPL U/mL	Negative < 10
Anti-cardiolipin IgM	1.6 MPL U/mL	Negative < 10
Anti-β2-glycoprotein I IgG	2.6 U/mL	Negative < 7
Anti-β2-glycoprotein I IgM	1.1 U/mL	Negative < 7
Lupus anticoagulant (DRVVT)	Positive	Negative

Note: The autoantibody profile, characterized by high-titer anti-SSA/Ro, anti-U1-RNP, and anti-SmD positivity, supports an overlap phenotype between systemic lupus erythematosus and Sjögren’s syndrome. ANA = Antinuclear Antibodies. IFA = Indirect Immunofluorescence Assay. EIA = Enzyme Immunoassay. DRVVT = Dilute Russell’s Viper Venom Time.

**Table 2:** Evolution of key laboratory parameters during hospitalization.

Parameter	Admission	Day 4	Second Febrile Episode	At SLE Diagnosis
Hemoglobin (g/dL)	10.7	10.3	11.4	8.5
Platelets (/mm^3^)	80,000	96,000	156,000	173,000
Leukocytes (/mm^3^)	8670	2410	2250	2180
CRP (mg/L)	135.9	29	7.6	2.6
ESR (mm/h)	71	NA	89	37
C3 (g/L)	0.64	NA	0.7	0.69
C4 (g/L)	0.08	NA	0.12	0.17
Proteinuria (g/24 h)	NA	NA	NA	0.68

CRP = C-reactive protein. ESR = Erythrocyte Sedimentation Rate. NA = Not Available.

## Data Availability

The original contributions presented in this study are included in the article. Further inquiries can be directed to the corresponding author.

## Abbreviations

The following abbreviations are used in this manuscript:

SLE	Systemic lupus erythematosus
SS	Sjögren’s syndrome
CT	Computed tomography
FESS	Functional endoscopic sinus surgery
